# Future Trends in Advanced Materials and Processes

**DOI:** 10.3390/ma15196554

**Published:** 2022-09-21

**Authors:** Petrica Vizureanu

**Affiliations:** 1 Faculty of Material Science and Engineering, Gheorghe Asachi Technical University of Iasi, 41 D. Mangeron St., 700050 Iasi, Romania; peviz@tuiasi.ro; 2 Materials Science and Engineering Department, Technical Sciences Academy of Romania, Bulevardul Dacia 26, 030167 București, Romania

The main objective of this Special Issue was to publish original high-quality research papers covering the most recent advances in materials properties, as well as comprehensive reviews addressing the relevant state-of-the-art topics in the area of materials processing, with relevant practical applications.

Nowadays, advanced materials and processes are available next to advanced characterization techniques. The special issue managed to gather several outstanding articles in a broad field, from non-metallic, concretes and porous materials to metallic biomaterials or molded products.

An important number of studies have shown that there are many differences in the expansibility of different dolostones, and the key factors determining the expansibility of alkali carbonate rocks. Some rocks were selected from five different geological ages: Jixianian, Cambrian, Ordovician, Devonian, and Triassic ages, and after determining the morphology of dolomites, it was concluded that there is a good positive correlation between ordering degree and the molar fraction of MgCO_3_ of dolomites [1]. The assessment of the radiological hazards associated with applying the investigated granite in the building materials for infrastructures application. Ref. [2] After classifying the granites, investigations were made related to the environmental parameters such as absorbed dose rate, annual effective dose, radium equivalent activity, and external and internal hazard indices. Main findings are that the investigated types of granites with high radioactivity concentration cannot be applied in the different applications of building materials and ornamental stone. Another radiological hazard evaluation of some egyptian magmatic rocks used as ornamental stone was made [3] and a number of nineteen samples were prepared from seven rock types for assessment in order to be used as ornamental stones. Using equipment as gamma-ray spectrometer or radiological hazard indices for natural radioactivity, the main conclusion was that the investigated rocks are suitable for use as ornamental stone in the construction buildings.

A novel process to recover gypsum from phosphogypsum [4] was achieved taking into consideration harmful elements, such as silicon, phosphorus, and fluorine. Gypsum was recovered using a direct flotation method and limited amounts of other minerals (muscovite, zoisite) were found in the gypsum concentrate during the flotation process because of mechanical displacement.

The effects of Niobium and Molybdenum on the microstructures and corrosion properties of high-entropy CrFeCoNiNb_x_Mo_x_ and CrFeCoNiNb_x_Mo_1−x_ alloys were investigated [5] by preparing in arc melting equipment under an argon atmosphere. Together with growing Nb and Mo percentages, it will increase the hardness of the alloys because of formation of the hexagonal close packing phase of the solid solution [6]. An interesting approach was to re-melt using a TIG welding process and to determine the behavior and wear resistance of Vanadium carbide precipitating Cr_27.5_Co_14_Fe_22_Mo_22_Ni_11.65_V_2.85_ High Entropy Alloy (HEA). The wear resistance was found to be similar to Stellite 6, which is a cobalt base alloy consisting of a matrix containing dispersed carbides.

Experimental results for thermal shock resistance and thermal insulation properties were obtained for lanthanum magnesium hexaluminate (LaMgAl_11_O_19_)/yttria-stabilised zirconia (YSZ) thermal barrier coating (FG-TBC) [7]. In the as-sprayed and laser-glazed conditions, microstructural changes were observed as a formation of cracks on coated surfaces. This procedure can significantly enhance the thermal performance of the coatings and can be used for different applications that require effective heat management (gas turbines). Moreover, transmission, reflection and dissipation of microwaves in magnetic composites were investigated for two compositions [8]. It was calculated the microwave losses and obtained the values for the dielectric permittivity and magnetic permeability. Another property investigated was electrical conductivity of a woven structure by describing a mixing model [9]. The woven structure was treated as a complex multiphase mixture being composed by two conducting phases and one non-conducting phase, with low connectivity in the conductive phases, as the results have shown. The main findings are regarding the strips’ contact phases that play an important role in the structure of the composite, also being very important is how the phases were arranged in the whole composite.

Another approach was developed by a comprehensive review, regarding the nonmetallic materials, which are introducing alkali activated based steel waste in the steelmaking industry [10]. The review focuses on the last ten years and is showing the most important providers of steel waste (blast, electric arc and basic oxygen furnaces), together with the possibilities of using the steel waste to improve the Ordinary Portland Cements properties through an alkali activation system. Another review on the same topic raises the issue of the innovative geopolymer composites designed for water and wastewater treatment [11]. The aim of the article is to make an assessment and to explain to the scientific world the main concerns in geo-synthesis, regarding the properties and applications of geopolymer composites that can be used for the elimination of hazardous contaminants. Moreover, the development of the self-compacting concrete mixtures based on some modifiers at a nanoscale was determined [12] by laboratory investigations and mathematical modelling applied to technological process and raw materials for concrete mixtures. In addition, mechanical properties of porous materials become important for various industries, such as energy storage [13]. The equivalent property of a material with pores was determined using a software and a comparison with measurement results were completed.

In the field of concretes and porous materials, an experimental development and mathematical dependencies of the gas release process in the production of non-autoclaved aerated were completed [14], trying to fill theoretical gaps in this phenomenon during the formation of the structure of aerated concrete. In this way, a new method to produce aerated concrete was published by early hydrating of the aerated concrete mixture, improving the gas-holding capacity. It was obtained via an eco-friendly material (aerated concrete) with better properties.

Titanium alloys were another focus of this special issue [15,16]; the main achievement was electrochemical impedance spectroscopy (EIS) characterization, since corrosion resistance is very important for this type of alloys. A new titanium alloy with addition of Ta in various percentages (5–30%) was investigated using scanning tunneling microscopy (STM) and energy dispersive X-ray spectroscopy (EDS). These binary alloys [15] can be considered for medical applications due to increasing of a passive layer resistance with the formation of the passive layer of TiO_2_ and Ta_2_O_5_. The obtaining of a new and promising titanium alloy [16] with 20% Molibdenum and various percentages of Sillicon (0.5 to 1.0) was followed by a characterization of the microsctructure, and it was investigated via their electrochemical responses in Ringer´s solution by linear polarization, cyclic potential dynamic polarization and EIS.

A different approach was discussed [17] in order to evaluate rapid tooling (RT) in rapid heat cycle molding (RHCM) by reviewing the implementation of this method in the molding industry. There are still limited studies available on molds fabricated using RT in RHCM to understand the mechanical properties and aesthetic properties (surface appearance) of the molded part, but RHCM represents an alternative to increase the quality of those products.

All this published researches will offer a new approach for future studies, in order to create important progresses in materials science and engineering. 

## References

[1] Fan Z., Mao Z., Liu X., Yi L., Zhang T., Huang X., Deng M. (2022). Microstructure of Dolostones of Different Geological Ages and Dedolomitization Reaction. Materials.

[2] El Dabe M.M., Ismail A.M., Metwaly M., Taalab S.A., Hanfi M.Y., Ene A. (2022). Hazards of Radioactive Mineralization Associated with Pegmatites Used as Decorative and Building Material. Materials.

[3] Lasheen E.S.R., Rashwan M.A., Osman H., Alamri S., Khandaker M.U., Hanfi M.Y. (2021). Radiological Hazard Evaluation of Some Egyptian Magmatic Rocks Used as Ornamental Stone: Petrography and Natural Radioactivity. Materials.

[4] Xiao J., Lu T., Zhuang Y., Jin H. (2022). A Novel Process to Recover Gypsum from Phosphogypsum. Materials.

[5] Tsau C.-H., Chen Y.-H., Tsai M.-C. (2022). The Effects of Niobium and Molybdenum on the Microstructures and Corrosion Properties of CrFeCoNiNbxMoy Alloys. Materials.

[6] Treutler K., Lorenz S., Wesling V. (2021). Re-Melting Behaviour and Wear Resistance of Vanadium Carbide Precipitating Cr27.5Co14Fe22Mo22Ni11.65V2.85 High Entropy Alloy. Materials.

[7] Anaz Khan M., Vivek Anand A., Duraiselvam M., Srinivas Rao K., Arvind Singh R., Jayalakshmi S. (2021). Thermal Shock Resistance and Thermal Insulation Capability of Laser-Glazed Functionally Graded Lanthanum Magnesium Hexaluminate/Yttria-Stabilised Zirconia Thermal Barrier Coating. Materials.

[8] Rinkevich A.B., Perov D.V., Ryabkov Y.I. (2021). Transmission, Reflection and Dissipation of Microwaves in Magnetic Composites with Nanocrystalline Finemet-Type Flakes. Materials.

[9] Tokarska M. (2022). A Mixing Model for Describing Electrical Conductivity of a Woven Structure. Materials.

[10] Aziz I.H., Abdullah M.M.A.B., Salleh M.A.A.M., Ming L.Y., Li L.Y., Sandu A.V., Vizureanu P., Nemes O., Mahdi S.N. (2022). Recent Developments in Steelmaking Industry and Potential Alkali Activated Based Steel Waste: A Comprehensive Review. Materials.

[11] Luhar I., Luhar S., Abdullah M.M.A.B., Razak R.A., Vizureanu P., Sandu A.V., Matasaru P.-D. (2021). A State-of-the-Art Review on Innovative Geopolymer Composites Designed for Water and Wastewater Treatment. Materials.

[12] Stel’makh S.A., Shcherban’ E.M., Beskopylny A., Mailyan L.R., Meskhi B., Beskopylny N., Zherebtsov Y. (2022). Development of High-Tech Self-Compacting Concrete Mixtures Based on Nano-Modifiers of Various Types. Materials.

[13] Pyo C., Kim Y., Kim J., Kang S. (2021). A Study to Derive Equivalent Mechanical Properties of Porous Materials with Orthotropic Elasticity. Materials.

[14] Shcherban’ E.M., Stel’makh S.A., Beskopylny A., Mailyan L.R., Meskhi B., Shuyskiy A., Beskopylny N., Dotsenko N. (2022). Mathematical Modeling and Experimental Substantiation of the Gas Release Process in the Production of Non-Autoclaved Aerated Concrete. Materials.

[15] Socorro-Perdomo P.P., Florido-Suárez N.R., Mirza-Rosca J.C., Saceleanu M.V. (2022). EIS Characterization of Ti Alloys in Relation to Alloying Additions of Ta. Materials.

[16] Baltatu M.S., Vizureanu P., Sandu A.V., Florido-Suarez N., Saceleanu M.V., Mirza-Rosca J.C. (2021). New Titanium Alloys, Promising Materials for Medical Devices. Materials.

[17] Huzaim N.H.M., Rahim S.Z.A., Musa L., Abdellah A.E.-h., Abdullah M.M.A.B., Rennie A., Rahman R., Garus S., Błoch K., Sandu A.V. (2022). Potential of Rapid Tooling in Rapid Heat Cycle Molding: A Review. Materials.

